# Birth after low-level +20 Aneuploid Mosaic Embryo Transfer: A Case Report

**DOI:** 10.5935/1518-0557.20230055

**Published:** 2024

**Authors:** Marta Ribeiro Hentschke, Aline Petracco Petzold, Isadora Badalotti-Teloken, Victória Campos Dornelles, Fabiana Mariani Wingert, Ricardo Azambuja, Maria Teresa Vieira Sanseverino, Alvaro Petracco, Mariangela Badalotti

**Affiliations:** 1 Fertilitat - Reproductive Medicine Center, Porto Alegre, RS, Brazil; 2 Pontifical Catholic University of Rio Grande do Sul, RS, Brazil

**Keywords:** blastocyst, trisomy, gestation, *in vitro* maturation, PESA, mosaic embryo transfer

## Abstract

**Objective:**

Recently, it has been discussed whether or not mosaic embryo transfers should be performed since they might result in viable pregnancies, although they often end up being discarded. We report a case of successful pregnancy, after a mosaic embryo transfer from an in vitro matured egg and frozen PESA sperm.

**Case Description:**

Tests performed on a female aged 40 years and a male aged 37 years seeking fertility treatment found she had an adequate ovarian reserve and patent fallopian tubes. He had a history of cryptorchidism and inguinal hernia repair. The spermogram showed azoospermia, and testicular ultrasound showed an atrophic left testicle and a normal right testis. The vas deferens was palpated during physical examination. Intracytoplasmic sperm injection with percutaneous epididymal sperm aspiration (PESA) was indicated. Two cycles of IVF after controlled ovarian stimulation with follitropin delta was performed. In the first cycle, seven mature eggs were inseminated, two fertilized normally, resulting in one blastocyst biopsied and analyzed by NGS with complex aneuploid results. In the second cycle, frozen sperm from PESA was used. Three eggs were inseminated on the day of the procedure (resulting in 2 blastocysts), and three in vitro matured eggs were inseminated after 24 hours (resulting in 1 blastocyst). NGS analysis showed two complex aneuploid embryos and one 40% low-level trisomy 20 aneuploid mosaicism (+20) for the post 24-hour embryo. A mosaic embryo transfer was performed, resulting in clinical pregnancy and birth of a healthy baby girl with a normal blood karyotype.

**Discussion:**

Mosaic embryo transfer is a topic for discussion. Certain levels of mosaicism do not seem to pose risks to the development of the fetus.

## INTRODUCTION

Low-level mosaic embryos present 30% to 50% of aneuploid cells (Abhari & Kawwass, 2021). Recently, it has been discussed whether or not mosaic embryo transfers (MET) should be performed, since they might result in viable pregnancies, although mosaic embryos often end up being discarded. Greco et al. (2015) reported healthy live births from mosaic monosomy embryos with mosaicism ranging from 35% to 50% affecting different chromosomes, although pregnancy outcomes of MET compared to euploid transfers were consistently lower. The primary concern regarding MET revolves around the factors that may affect live birth rate, such as the prenatal and postnatal health of babies derived from mosaic embryos. The viable and karyotypically normal live births by MET post it as an option for patients with no euploid embryo produced after assisted reproduction (Zhang et al., 2020). The last statement of the Preimplantation Genetic Diagnosis International Society - PGDIS (2021) considered MET as a safe option for couples, with low or minimal risk of negative outcomes for the birth (Leigh et al., 2022).

Oocyte in vitro maturation after stimulated cycles is a possibility when there is not a considerable number of mature oocytes for fertilization. However, it can result in lower pregnancy rates (Reichman et al., 2010).

This study reports a case of successful pregnancy after mosaic embryo transfer from an in vitro matured egg (from metaphase I to metaphase II) and frozen PESA sperm. We hope to bring further elucidation on the topic of mosaic embryo transfer.

## CASE DESCRIPTION

Tests performed on a 40-year-old female and a 37-year-old male patient had the following results: follicle-stimulating hormone (FSH): 6.3 mIU/ml; estradiol (E2): 2.9 ng/dL; anti-Müllerian hormone (AMH): 1.27ng/ml; antral follicle count (AFC): 15 in both ovaries. She presented normal pelvic anatomy and patent tubes. He had a history of cryptorchidism and contralateral inguinal hernia repair; his seminal analysis results were: volume: 2.2 mL, round cells: 50,000/mL, azoospermia, which was confirmed through a second examination with an interval of three months. His ultrasound showed an atrophic left testicle and a normal right testis; the vas deferens was palpated during physical examination. The couple was prescribed intracytoplasmic sperm injection (ICSI) with percutaneous epididymal sperm aspiration (PESA).

The couple underwent two cycles of in vitro fertilization (IVF). The embryos were cultured in an embryoscope. Hatching was performed on day three of embryo development, and biopsy was performed on day five. Around 5-10 trophectoderm cells were obtained from biopsy; they were placed in a small reaction tube for amplification and testing in the genetics lab.

The first IVF cycle was carried out with controlled ovarian stimulation with a total of 136 mcg of follitropin delta and PESA (various motile and immobile sperm). Seven mature eggs were inseminated, two were fertilized normally, resulting in one blastocyst biopsied and analyzed with next generation sequencing (NGS), showing a complex aneuploid result: +3, -4, +6, +11, -18.

The second cycle, with a total of 128 mcg of follitropin delta and frozen sperm from PESA, resulted in three mature eggs (MII) inseminated on the day of the procedure, and five immature oocytes placed in culture medium and left in the incubator for 24 hours. All three MII oocytes fertilized, and from the previous five immature eggs, four were inseminated and three fertilized, resulting in six embryos being cultured. Two blastocysts were obtained from fertilized eggs on the day of the procedure, and one blastocyst from eggs inseminated after 24 hours. Genetic analysis was carried out, showing two complex aneuploid embryos (the first one +15, -18, -19, +20, +21, and the other one +3, +11, +20, -22), and a low-level trisomy 20 aneuploid mosaicism for the post 24-hour embryo. After genetic counseling with a geneticist specialized in infertility, a mosaic embryo transfer was performed, resulting in pregnancy.

The patient had chronic hypertension, which was treated during pregnancy with methyldopa 250 mg, 3 times a day; prophylaxis for preeclampsia was performed with enoxaparin 40 mg per day and acetylsalicylic acid 100 mg per day.

The patient had normal first and second trimester findings in morphological ultrasound; the placenta had a good flow, though bilobed. As for complications during pregnancy, in the third trimester, the fetus weight percentile was relatively low; the patient was prescribed relative rest.

A female newborn was delivered through a cesarean section due to breech presentation at 38 weeks and 4 days of pregnancy. She was born in good condition, with no complications, measuring 48 cm and weighing 3200 g. The 1-minute Apgar score was 9 and the 5-minute Apgar score was 10. Neonatal screening tests (blood, red reflex, hearing, and cardiac screening) were normal. At two months of age, the baby is growing and developing normally, reaching every expected milestone; she is in the 50th percentile for her age. The karyotype of the baby girl, derived from peripheral blood analysis, was normal (46,XX) and no structural abnormalities were found.

Thus, this case showed a successful transfer of a low-level mosaic embryo, indicating the possibility of successful pregnancy after MET.

## DISCUSSION

Mosaic embryo transfer (MET) has been a topic of discussion, with limited information regarding outcomes published in the literature. Although certain levels of mosaicism do not seem to pose risks to the safe development of the fetus, some authors worry about the potential long-term implications of MET, which have not yet been thoroughly documented. In the case reported herein, the patient had normal first and second trimester morphological ultrasound scans, and delivered a healthy newborn.

Recent studies that compared MET with euploid embryo transfers found no difference in birth weight, preterm delivery rate, or risk of congenital malformations (Abhari & Kawwass, 2021). In the case described in this report, the newborn had adequate birth weight after a term pregnancy, with no congenital malformations. However, growing evidence suggests that MET is associated with lower implantation rates and higher risk of miscarriage (Abhari & Kawwass, 2021), which was not noted in our patient’s case.

The majority of the experts agree that MET should only be performed in situations in which no euploid embryos are available for transfer, and after thorough discussions with the family about the implications of the procedure and alternative options. Moreover, MET should be followed by comprehensive genetic counseling, emphasizing on prenatal diagnostic testing, in order to prepare and enlighten the family on potential future outcomes (Franasiak, 2022; Surrey, 2021). In the reported case, considering the advanced maternal age and male infertility, and the fact that after two IVF cycles only aneuploid or mosaic embryos were available for transfer, MET was performed. Genetic analysis and counseling were carried out, so that the patients were well aware and prepared for possible adverse outcomes.

Following ovarian stimulation, oocytes are retrieved at varying stages of meiotic maturity, being, occasionally, retrieved at the germinal vesicle or metaphase I stage. Over the last years, much research has focused on *in-vitro* maturation of immature oocytes retrieved after stimulated cycles, and successful pregnancies from extended incubation have been reported in the literature, albeit with low incidence. More recent studies have indicated that such oocytes give rise to embryos with compromised developmental characteristics and very low implantation potential, with greater detrimental effects in parameters such as cell number, symmetry, and fragmentation (Reichman et al., 2010). Thus, we highlight the fact that our patient presented a successful pregnancy after *in vitro* maturation of oocytes after a previously stimulated cycle.

Furthermore, recent studies have indicated that the frequency of aneuploidy and mosaicism in embryos obtained from men with azoospermia is very high, regardless of the age of their female partners. An explanation for this high rate might be a compromised testicular environment, or that abnormal spermatozoids are usually sequestrated along the way between the testis and the tip of the urethra; thus, we would be comparing selected (through the epididymis and prostate) with non-selected sperm (Platteau et al., 2004). Azoospermia presented by our patient could, therefore, explain the number of aneuploid or mosaic embryos obtained.

Finally, we emphasize that the overall success of human reproduction, spontaneously or after IVF, highly depends onn maternal age. The main reasons for age-related infertility include reduced ovarian reserve and decreased oocyte/embryo competence due to aging resulting in increased incidence of aneuploidies. Age-related chromosomal abnormalities mainly arise because of meiotic impairments during oogenesis, following flawed chromosome segregation patterns such as non-disjunction, premature separation of sister chromatids, or reverse segregation (Cimadomo et al., 2018). Our patient was 40 years old, which might have contributed to the number of aneuploid and mosaic embryos obtained.

In conclusion, we stress the importance of adopting a standardized approach to the transfer of embryos diagnosed with secondary findings such as mosaicism, which should include thorough genetic counseling on the specific and possible outcomes of mosaicism. Moreover, it is also relevant to be wary of the higher rates of aneuploidy and mosaicism in female patients with advanced age and in male patients with azoospermia - cases in which more thought should be given to transferring mosaic embryos. Still, further studies focusing on perinatal and long-term outcomes of children born after MET are needed to elucidate the potential long-term implications of this procedure.
